# Supplementary material for: Role of Microtubule-Associated Protein 1b in Urothelial Carcinoma: Overexpression Predicts Poor Prognosis
Source: Cancers (Basel). 2020 Mar 9;12(3):630. doi: 10.3390/cancers12030630 (PMC7139768; doi:10.3390/cancers12030630)
Supplement: Supplementary file 1 [file cancers-12-00630-s001.pdf]

Supplementary Materials

Role of Microtubule-Associated Protein 1b in Urothelial Carcinoma: Overexpression Predicts Poor Prognosis

Tsu-Ming Chien, Ti-Chun Chan, Steven Kuan-Hua Huang, Bi-Wen Yeh, Wei-Ming Li, Chun-Nung Huang, Ching-Chia Li, Wen-Jeng Wu and Chien-Feng Li

Table S1. Urothelial carcinoma enrolled to explore potential MAP1B mutation.

Case No.	Age (year)	Gender	Tumor location	pT	pN
UBUC-1	78	M	Urinary bladder	T1	N0
UBUC-2	54	M	Urinary bladder	T1	N0
UBUC-3	56	M	Urinary bladder	T2	N0
UBUC-4	69	M	Urinary bladder	T3	N1
UBUC-5	74	F	Urinary bladder	T1	N0
UBUC-6	91	F	Urinary bladder	T2	N0
UBUC-7	66	M	Urinary bladder	T1	N0
UBUC-8	61	F	Urinary bladder	T3	N0
UBUC-9	69	F	Urinary bladder	T2	N0
UBUC-10	56	F	Urinary bladder	T2	N2
UBUC-11	85	M	Urinary bladder	T3	N2
UBUC-12	85	M	Urinary bladder	T3	N2
UBUC-13	59	M	Urinary bladder	T1	N0
UBUC-14	81	F	Urinary bladder	T2	N0
UBUC-15	56	M	Urinary bladder	T3	N0
UTUC-1	72	F	R’t pelvis & ureter	T2	N0
UTUC-2	53	F	L’t pelvis	T3	N0
UTUC-3	69	F	R’t pelvis & ureter	T1	N0
UTUC-4	60	M	L’t ureter	T3	N0
UTUC-5	60	M	L’t pelvis	T4	N1
UTUC-6	67	M	R’t pelvis	T3	N0
UTUC-7	57	F	R’t ureter	T3	N1
UTUC-8	61	F	L’t pelvis & ureter	T3	N0
UTUC-9	75	M	R’t ureter	T3	N0
UTUC-10	87	F	R’t pelvis	T4	N2
UTUC-11	65	F	L’t pelvis & ureter	T2	N0
UTUC-12	64	F	L’t pelvis & ureter	T1	N0
UTUC-13	63	F	L’t pelvis	T3	N1
UTUC-14	63	M	R’t ureter	T3	N0
UTUC-15	68	M	R’t pelvis	T3	N2

Table S2. MAP1B mutations validated and primer sets.

Amino acid change	Ref. Study	Type	Primers	Product size
S2074L	Bladder (BGI 2013)	Missense	Forward: CAGGCATCCACATATTCCTACG<br>Reverse: TGAGGGGCTTTTCAGATTCTTC	214 bp
S1280T	Bladder (DFCI/MSKCC 2014)	Missense	Forward: AAGTGAAAGCCAGCACCACTTT<br>Reverse: CACCACTTCCAGAGTCTTGTCC	275 bp
K1476N	Bladder (TCGA 2014)	Missense	Forward: GCCGAAAGTCCTTTTGAAGAAA<br>Reverse: TGACATCTATTTGTGTGGGAGAAA	250 bp
S1692C	Bladder (TCGA 2014)	Missense	Forward: AATTTGGCCAAGAATCTCCTGA<br>Reverse: GTGTAGGACGGCTCCTCCATAG	193 bp
E1129K	Bladder (TCGA 2014)	Missense	Forward: GTATGGATTCCTCACCACACCA<br>Reverse: GGGACTCCGTCTCTTCATTGTT	281 bp
G287R	Bladder (TCGA 2014)	Missense	Forward: CCCTTTGACATCTTGGAACCTC<br>Reverse: CACTCGGTCTAAGTGTCGGATG	189 bp
E1097K	Bladder (TCGA 2014)	Missense	Forward: GTATGGATTCCTCACCACACCA<br>Reverse: GGGACTCCGTCTCTTCATTGTT	281 bp
Q1873*	Bladder (TCGA 2014)	Nonsense	Forward: AATGCAACACCATCTAGCCTTG<br>Reverse: GTCCGGGTGGTCTTCTCATAA	174 bp

Table S3. Characters of independent UBUC patient cohorts receiving postoperative adjuvant chemotherapy.

Parameter	Category	Case No.
**Gender**	Male	56
	Female	14
**Age (years)**	39–88 (mean: 69)	70
**Primary tumor (T)**	T3	56
	T4	14
**Nodal metastasis**	Negative (N0)	50
	Positive (N1–N2)	30

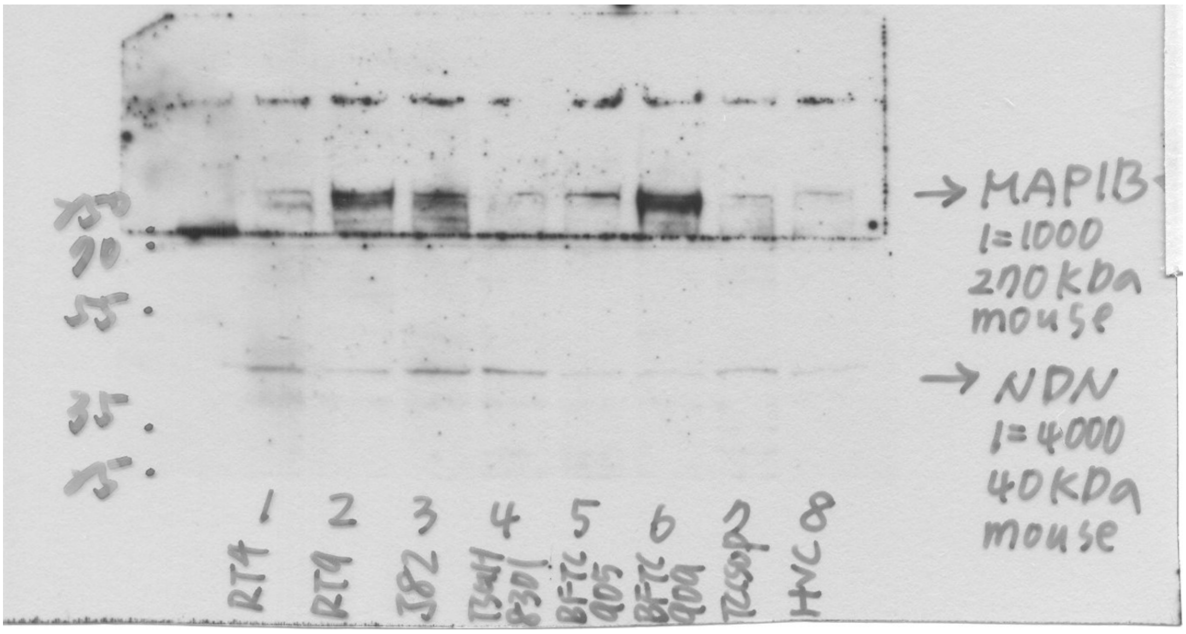

Western Blot.

© 2020 by the authors. Licensee MDPI, Basel, Switzerland. This article is an open access article distributed under the terms and conditions of the Creative Commons Attribution (CC BY) license (http://creativecommons.org/licenses/by/4.0/).
